# Tetraspanin 3 promotes NSCLC cell proliferation via regulation of β1 integrin intracellular recycling

**DOI:** 10.1186/s11658-024-00639-w

**Published:** 2024-09-27

**Authors:** Yao Zhang, Chenglong Wang, Yitong Xu, Hongbo Su

**Affiliations:** 1 Department of Pathology, the First Hospital and Basic Medical Sciences College of China Medical University, Shenyang, 110001 China; 2 Department of Pain, the First Hospital of China Medical University, Shenyang, 110001 China; 3 Department of Pathology, the First Hospital of China Medical University, No. 155 Nanjing North Street, Heping District, Shenyang, 110001 Liaoning China

**Keywords:** β1 integrin, Tetraspanin 3, Non-small cell lung carcinoma, Rab11a

## Abstract

**Background:**

The involvement of tetraspanins in cancer development has been widely implicated. In this study, the function and molecular mechanisms of tetraspanin 3 (TSPAN3) in non-small cell lung cancer (NSCLC) cells were explored.

**Methods:**

Tissue samples from patients diagnosed with NSCLC were analyzed by immunohistochemistry, western blotting, and real-time polymerase chain reaction (PCR) to indicate the involvement of TSPAN3 in cancer progression. In the meantime, we also performed exhaustive mechanistic studies using A549 and H460 cells in vitro through a variety of methods including western blotting, real-time PCR, immunofluorescent staining, coimmunoprecipitation, cell proliferation assay, and nocodazole (NZ) washout assay. Proper statistical analysis was implemented wherever necessary in this study.

**Results:**

TSPAN3 was found to be highly expressed in lung cancer cells and tissues. Moreover, high levels of TSPAN3 positively correlated with poor differentiation, lymph node involvement, advanced pathological tumor-node-metastasis stage, and poor prognosis in patients with NSCLC. TSPAN3 showed potential to promote the proliferation of NSCLC cells in vitro and in vivo. Specifically, TSPAN3 was found to interact with β1 integrin via the LEL domain, thereby facilitating the sorting of β1 integrin into Rab11a endosomes and promoting β1 integrin recycling and upregulation.

**Conclusions:**

Our findings reveal TSPAN3 may represent a potentially valuable therapeutic target for NSCLC.

**Supplementary Information:**

The online version contains supplementary material available at 10.1186/s11658-024-00639-w.

## Introduction

The trafficking of integrins through the endosomal pathway is an underexploited target of next generation anticancer agents due to limited understanding of its role in integrin function and cancer progression [1, 2].

Cell surface integrins undergo continuously endocytosis-surface cycles [3, 4]. Previous studies have shown that integrins are transported to the early endosomes after endocytosis, where sorting decisions are made as to whether the endocytosed integrins will be transferred to late endosomes and lysosomes for degradation, or they will be recycled through one of two distinct routes. Instead of being degraded, most integrins return to the cell membrane through the Ras-related protein 4 (Rab4)-mediated route (also called the short loop) or move to the perinuclear recycling compartment and are transported to the cell membrane through the Rab11‑mediated route (also called the long loop) [4–7]. Although the understanding of integrin trafficking is rapidly expanding, the mechanism through which cells select a specific trafficking route for different integrins remains unclear. Importantly, the mediatory role of Rabs in the trafficking of specific integrins requires further investigation [8].

Tetraspanins is a family of proteins embedded in the membrane through four transmembrane domains. Albeit their abundance in most eukaryotes the biological functions of tetraspanins have not been studied extensively [9]. These proteins contain two extracellular domains: the large extracellular loop (LEL) domain and small extracellular loop (SEL) domain. The LEL domain was reported to be crucial for the interaction of tetraspanins with multiple membrane proteins such as β1 integrins. Indeed there is accumulating evidence emerged in recent years to indicate the crucial role of tetraspanins in regulating the function and trafficking of membrane proteins. For example, downregulation of tetraspanin-24 (CD151) significantly inhibits the endocytosis of α3β1 integrin in cells seeded on laminin-5 [10]. Similarly, the expression of surface CD19 has been reported to be downregulated in tetraspanin-28 (CD81)^−^ cells, suggesting a regulatory role for CD81 in CD19 trafficking. Further analysis suggested that the transport rate of CD19 from the endoplasmic reticulum to the Golgi compartment was significantly slower in CD81^−^ cells than in CD81^+^ cells [11, 12].

Tetraspanin 3 (TSPAN3) belongs to the tetraspanin superfamily. Despite limited studies on the biological function of TSPAN3, its potential role in cell proliferation via regulating integrin has come to our attention: TSPAN3 was reported to form a complex with β1 integrin in oligodendrocytes in neural cells [13]. Moreover, overexpression of TSPAN3 was reported to promote the proliferation of mouse oligodendrocyte cells [14, 15]. On the other hand, TSPAN3 harbors the YXXΦ tyrosine-based sorting motif, which has been widely reported to be associated with endocytosis or trafficking [16, 17], such as the recycling of β2 integrins [18].

This study aims to explore *TSPAN3* expression and clinicopathological significance in non-small cell lung carcinoma (NSCLC) to determine its potential role in cancer progression. Additionally, based on previous research, the study further aims to investigate the regulatory effect of TSPAN3 on β1 integrin through tuning intracellular trafficking in the context of NSCLC.

## Methods

Our study examined male and female, and similar findings are reported for both sexes.

### Patients and specimens

A total of 105 tissue samples from patients diagnosed with NSCLC between 2013 and 2015 and corresponding clinicopathological information were acquired from the Pathology Department of the First Affiliated Hospital of China Medical University with signed informed consent. All tumors were collected through curative surgical resection. None of the enrolled patients had received presurgical chemotherapy or radiation. Among the 105 tissue samples, 81 were paraffin-embedded tissues for immunohistochemistry analysis, whereas 24 tissues were fresh samples, of which 8 and 16 samples were used for western blotting and real-time polymerase chain reaction (RT-PCR) analyses, respectively. All patients were enrolled into the study retrospectively and randomly. The study was approved by the Medical Research Ethics Committee of the First Affiliated Hospital of China Medical University [KLS (2023) 221]. The ethics approval also included the informed consent exemption. All 81 patients whose samples were used for immunohistochemistry analysis were followed up for at least 3 years.

### Cell lines and cell culture

The human bronchial epithelial (HBE) cell line was obtained from the American Type Culture Collection (ATCC; Manassas, VA). The other cell lines (A549, H460, H1299, H1975, H226, H661, 293 T, and SK-MES-1) used were purchased from the Shanghai Cell Bank (Shanghai, China). A549, H1299, H460, H661, H266, and H1975 cells were cultured in RPMI-1640 medium, SK-MES-1 cells in minimal essential medium, and HBE and 293 T cells in Dulbecco’s modified Eagle medium, respectively. The media for H1975 cell line were supplemented with 20% fetal bovine serum (FBS), while all other media with 10% FBS. All media were purchased from Gibco (Waltham, MA), and FBS was purchased from Clark Bioscience (Webster, TX).

### Immunohistochemistry (IHC)

As described previously [19], tissue sections were probed with appropriate primary antibodies (Supplementary Table 1). Staining intensity was scored as follows: 0 (no staining), 1 (weak), 2 (moderate), or 3 (high). Percentage scores were assigned as follows: 1 (0–25%), 2 (26–50%), 3 (51–75%), and 4 (76–100%). The final multiplicative score for each specimen (0 − 12) was calculated by multiplying the intensity score with the percentage score. Tumor tissues with scores > 6 were considered as positive expression, whereas those with scores ≤ 6 were considered as negative expression.

### Cell treatment and transfection

Lipofectamine 3000 (Invitrogen, Waltham, MA) was used for transfection according to the manufacturer’s instruction. Information on siRNAs and plasmids is provided in Supplementary Materials.

Nocodazole (NZ) washout was performed to explore β1 integrin trafficking as previously described [20]. After 12 h of serum starvation, A549 cells were treated with NZ (10 µM, no. 31430-18-9, MedChemExpress, Monmouth Junction, NJ) for 4 h to completely depolymerise the microtubules (MTs). Then, the drug was washed off with serum-free medium to allow MT repolymerisation at different intervals after NZ washout.

For fibronectin (FN) stimulation of cells, recombinant human FN (10 μg/mL, no. 1918-FN-02 M, R&D Systems, Minneapolis, MN) was added to RPMI-1640 medium containing 1% FBS.

### Western blotting

Whole cell lysates were prepared from cells and tumor tissues using the NP-40 lysis buffer (P0013F, Beyotime Biotechnology, Shanghai, China) containing PMSF (1:100, ST506; Beyotime Biotechnologya) and phosphatase inhibitor (1:100, B15002; Biotool, Shanghai, China). Total protein was quantified using the Bradford method, 60 µg protein of each sample resolved with 10% sodium dodecyl–sulphate polyacrylamide gel electrophoresis and transferred onto polyvinylidene difluoride membranes (Millipore, Burlington, MA). The membranes were then probed overnight with the appropriate primary antibodies at 4 °C (Supplementary Table 1), washed three times with Tris-buffered saline and Tween 20 (TBST) buffer, and incubated with horseradish peroxidase-conjugated anti-mouse or anti-rabbit secondary antibodies (1:2,000; Proteintech) at room temperature for 2 h. The protein bands were visualized via enhanced chemiluminescence (H34080; Thermo Fisher Scientific) using a BioImaging System (UVP, Upland, CA). Relative expression was analyzed using ImageJ (National Institutes of Health, Washington, DC); the expression of target proteins was normalized against that of GAPDH or β-actin.

### Quantitative RT-PCR

Total RNA of cells and tissues were extracted as described previously [21]. Quantitative RT-PCR was carried out in a 7900HT Fast Real-Time PCR System (Applied Biosystems, Foster City, CA) using SYBR Premix Ex Taq II (RR820A, Takara Bio, Beijing, China) in a total volume of 20 μL according to the manufacturer’s instruction. Relative gene expression was calculated by the ΔΔCt method; the β-actin-encoding gene was used as reference. The primer sequences used are provided in Supplementary Materials. All experiments were performed in triplicate.

### Cell proliferation assay

Cells transfected with the TSPAN3-expressing plasmid or empty vector were seeded (3000 cells/well) in 96-well plates for MTS cell proliferation assay. Absorbance at 450 nm was detected every 24 h. A growth curve was generated using the absorbance values captured over 4 days of culture.

### Colony formation assay

Cells transfected with TSPAN3-expressing plasmid or empty vector were seeded (500 cells/well) in six-well plates and cultured until the formation of visible colonies. Images were acquired on a bio-imaging system (DNR, Neve Yamin, Israel). All experiments were performed independently at least three times.

### Coimmunoprecipitation and mass spectrometric assay

Assays were performed as previously described [22].

### Immunofluorescence

The A549 and H460 cells were seeded in 24-well (2 × 10^5^ cells) plates and cultured for 24 h, fixed with 4% paraformaldehyde for 10 min, blocked with 5% bovine serum albumin for 2 h, and then probed overnight with the appropriate primary antibodies at 4 °C (Supplementary Table 1). After washing three times with PBS, the cells were incubated with TRITC-conjugated or FITC-conjugated secondary antibodies at room temperature for 2 h, and the nuclei were stained with 4′,6-diamidino-2-phenylindole (DAPI; C1005, Beyotime Biotechnology). Images were captured using an Olympus FV3000 laser-scanning confocal microscope (Olympus, Tokyo, Japan).

For quantification of β1 integrin and Rab11a colocalization, Pearson correlation coefficient was analyzed through Fluoview software (FV31S-SW, Olympus, Tokyo, Japan).

### Flow cytometry

A549 cells collected via trypsinisation at different time points after NZ washout were washed with cold PBS. Resultant cells (1 × 10^5^ cells/sample) were stained with PE-conjugated β1 integrin antibody (1:100 no. 12-0299-42; eBioscience, San Diego, CA) at 4 °C for 20 min. The samples were washed three times to remove unbound antibodies and fixed with cold 4% paraformaldehyde for 20 min. The mean fluorescence intensity of each sample was measured using a FACScan flow cytometer (BD, Franklin Lakes, NJ).

### Tumor formation in nude mice

The process is described in Supplementary Materials.

### Proliferation Edu assay

The process is described in Supplementary Materials.

### Statistical analyses

The relationships between TSPAN3 expression and clinicopathological factors and d β1 integrin were statistically analyzed and tested using the chi-squared test. The association between TSPAN3 expression and the prognosis of patients with NSCLC was analyzed using both Kaplan–Meier and Cox proportional hazards models. All the statistical analyses were performed using SPSS 17.0 software (SPSS, Inc., Chicago, IL). Differences between two groups were analyzed through paired Student’s *t*-test using Prism 6.0 (GraphPad Software, San Diego, CA). *P* values < 0.05 were considered significant.

## Results

### High expression of TSPAN3as a prognostic factor in NSCLC may predict poor patient survival

**Fig. 1:**
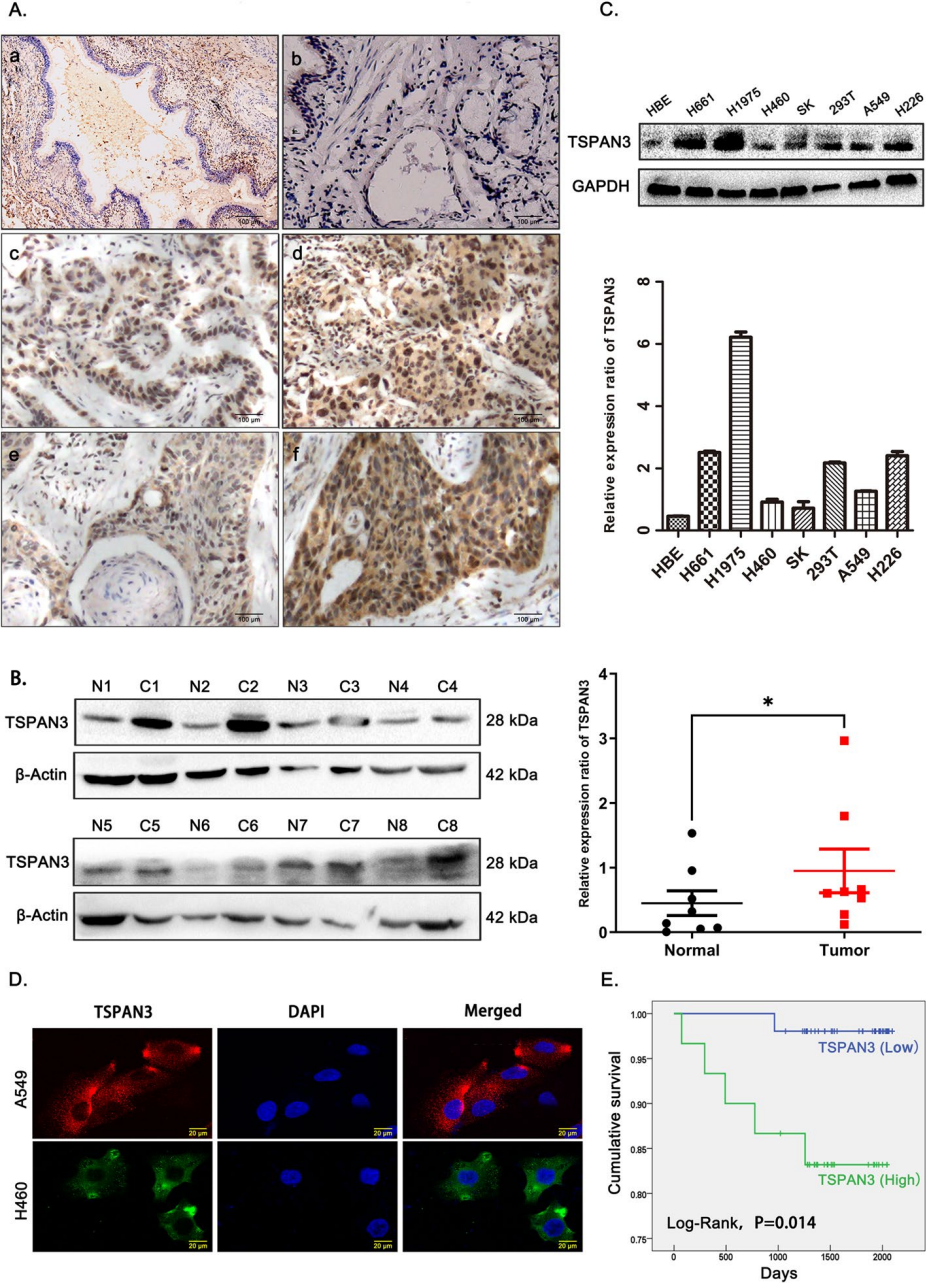
Expression pattern of TSPAN3 in NSCLC tissues and cell lines. **A** Tetraspanin 3 (TSPAN3) is expressed at low levels in normal bronchial epithelial cells (a) and submucosal gland cells (b) and expressed at high levels in NSCLC cells, well-differentiated adenocarcinoma (c), poorly differentiated adenocarcinoma (d), well-differentiated squamous carcinoma (e), and poorly differentiated squamous carcinoma (f). Magnification: × 400. **B** Western blot showing that TSPAN3 levels in NSCLC tissues (**C**) are higher than those in the surrounding normal tissues (N). β-actin served as the loading control. Relative protein expression was analyzed using ImageJ. *P < 0.05. **C** The endogenic TSPAN3 levels in six lung cancer cell lines, HBE cell line, and 297 T cell line as measured by western blotting and analyzed using ImageJ, GAPDH served as the loading control (*n* = 3 independent experiments). **D** Immunofluorescent probing indicates cytoplasmic localization of TSPAN3 in A549 and H460 cells. **E** Patients with lung cancer with high expression (IHC scores > 6) of TSPAN3 had shorter survival periods than those with low expression (IHC scores ≤ 6) of TSPAN3 (*P* = 0.014)

Immunohistochemistry and immunofluorescence analyses were performed to assess the expression of TSPAN3 in 81 NSCLC tissues and 2 NSCLC cell lines. TSPAN3 was found to be mainly localized in the cytoplasm (Fig. 1A, D). In addition, significantly higher levels of TSPAN3 were observed in NSCLC tissues than in the normal bronchial epithelium and submucosal glands (Fig. 1A). Western blotting was performed to assess TSPAN3 expression in eight pairs of fresh NSCLC tissues and adjacent normal tissues. NSCLC specimens were found to express prominently higher levels of TSPAN3 than their paired normal tissues (Fig. 1B). Baseline expression of TSPAN3 was also examined in six human lung cancer cell lines along with HET293T and HBE cell lines. TSPAN3 levels were consistently higher in all six lung cancer cells than those in HBE cells (Fig. 1C). Statistical analysis suggested that high TSPAN3 expression in NSCLC was positively associated with poor differentiation, lymph node involvement, and advanced pathological tumor-node-metastasis stage (*P* = 0.006, 0.006, 0.011, respectively; Table 1) but showed no significant association with sex, age, and histological type (*P* > 0.05; Table 1). Kaplan–Meier survival analysis, after following up all 81 patients for at least 3 years, further revealed that the survival period of patients with high TSPAN3 levels [IHC scores > 6, 1800.2 days, 95% confidence interval (CI) 1595.3–2005.4] was significantly shorter than that of patients with low TSPAN3 levels (IHC scores ≤ 6, 2072.8 days, 95% CI 2029.8–2115.9) (*P* = 0.014; Fig. 1E). Cox analyses also revealed that positive TSPAN3 expression (*P* = 0.043; hazard ratio of 9.139, 95% CI 1.067–78.248; Supplementary Table 2) was an independent prognostic factor in NSCLC.

**Table 1:** Correlation of tetraspanin 3 (TSPAN3) expression with clinical and pathological characteristics of patients with NSCLC

Correlation parameters					
Characteristics	*N*	Negative	Positive	*χ*^2^	*P*
Sex					
Male	51	32	19	0.003	0.958
Female	30	19	11		
Age (years)					
≤ 59	38	27	11	2.009	0.156
> 59	43	24	19		
Histology					
Squamous-cell carcinoma (SCC)	28	18	10	0.032	0.858
Adenocarcinoma (AC)	53	33	20		
Differentiation					
Well	24	20	4	10.156	0.006
Moderate	30	20	10		
Poor	27	11	16		
Nodal status					
No	50	39	11	7.756	0.006
Yes	31	12	19		
Tumor node metastasis (TNM) stage					
I + II	64	45	19	7.063	0.011
III + IV	17	6	11		

### TSPAN3 interacts with β1 integrin via its LEL domain and upregulates β1 integrin expression in NSCLC cells.

**Fig. 2:**
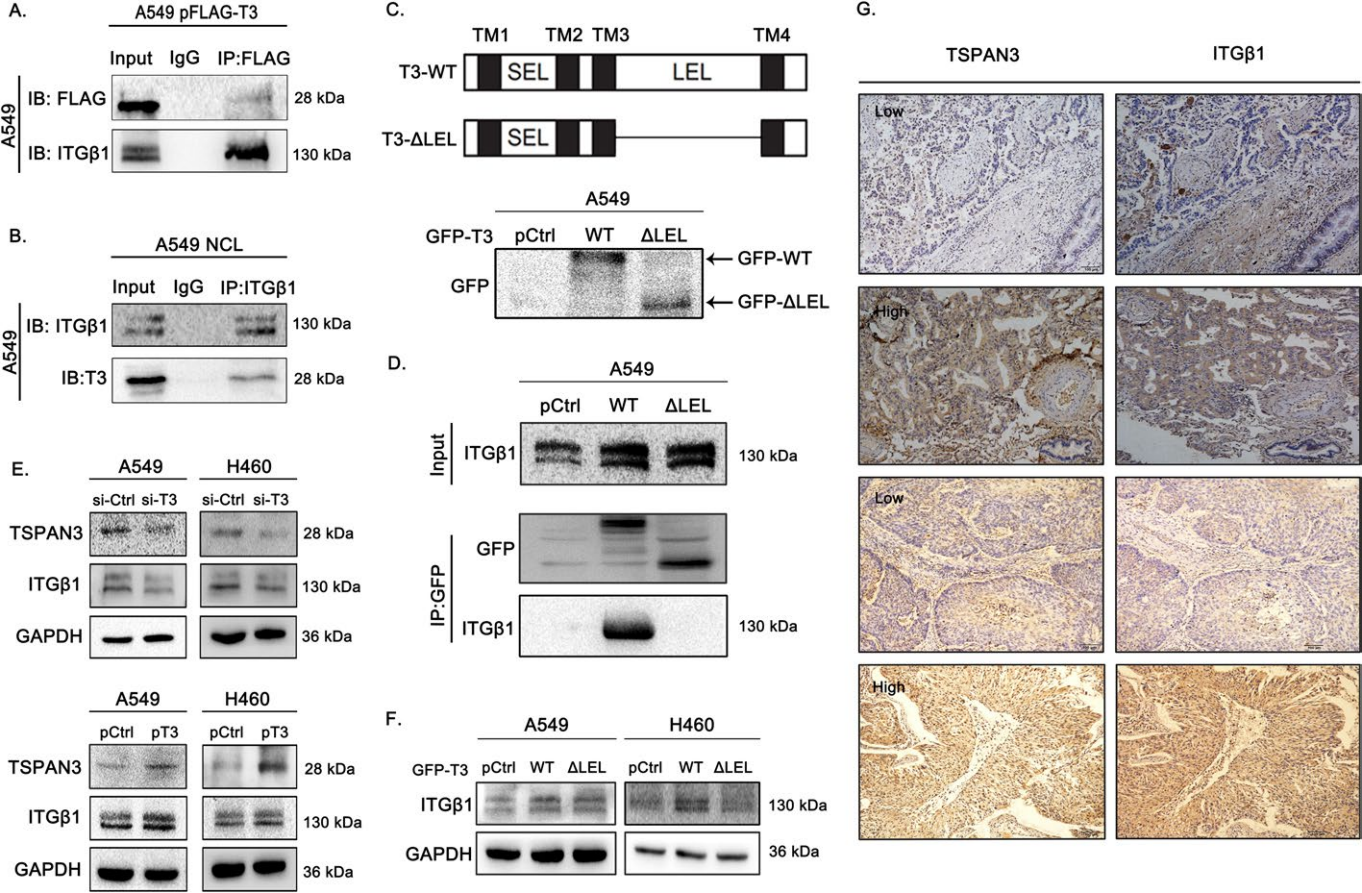
TSPAN3 interacts with—and upregulates—β1 integrin. **A** Ectopically expressed tetraspanin 3 (TSPAN3/T3) interacts with β1 integrin in A549 cells. 48 h post transfection of pCMV6-Myc-FLAG-TSPAN3 plasmid, cell lysates were immunoprecipitated with anti-FLAG antibodies or control IgG and then subjected to western blotting with anti-β1 integrin and anti-TSPAN3 antibodies. **B** Coimmunoprecipitation revealed that endogenous TSPAN3 interacts with β1 integrin in A549 cells. Cell lysates were immunoprecipitated with anti-TSPAN3 antibody or control IgG and then subjected to western blotting using anti-β1 integrin (ITGβ1) and anti-TSPAN3 antibodies. **C** Schematic of TSPAN3 LEL domain deletion. **D** Immunoprecipitation study indicates that TSPAN3 interacts with β1 integrin via the LEL domain. A549 cells were transfected with wild-type (WT) GFP-TSPAN3 or TSPAN3-ΔLEL mutant plasmids. After 48 h of transfection, cell lysates were immunoprecipitated with the anti-GFP antibody; the presence of β1 integrin was measured by western blotting with the anti-β1 integrin antibody. **E** Western blotting shows that TSPAN3 knockdown downregulated the expression of β1 integrin in A549 and H460 cells. Overexpression of TSPAN3 had the opposite effect. GAPDH served as the loading control (*n* = 3 independent experiments). **F** Overexpression of wild type TSPAN3 resulted in upregulated expression of β1 integrin, whereas overexpression of the TSPAN3-ΔLEL mutant did not exhibit any such effect. GAPDH served as the loading control (*n* = 3 independent experiments).**G** The association between TSPAN3 and β1 integrin investigated using immunohistochemistry (magnification ×200). IB, immunoblotting; IP, immunoprecipitation

Coimmunoprecipitation was performed in A549 cells to investigate whether TSPAN3 could interact with β1 integrin. Both endogenous and transfected TSPAN3 showed efficient interaction with β1 integrin (Fig. 2A, B). As LEL domain plays a vital role in mediating the interaction of TSPAN3 with other membrane proteins, a TSPAN3 LEL-deleted construct was generated (Fig. 2C) to further explore the dependency of TSPAN3 and β1-integrin interaction on LEL domain. Coimmunoprecipitation revealed that the interaction between TSPAN3 and β1-integrin was abrogated when LEL domain was deleted (Fig. 2D). Next, to investigate the regulation of β1 integrin by TSPAN3, A549 and H460 cells (cells with moderate TSPAN3 expression) were transfected with a TSPAN3-expressing plasmid. TSPAN3 overexpression upregulated β1 integrin levels relative to the control, whereas TSPAN3 knockdown showed the opposite effect (Fig. 2E). A549 and H460 cells were also transfected with plasmids harboring the wild-type or mutant (LEL deleted; TSPAN3-ΔLEL) TSPAN3. Western blotting showed that overexpression of wild-type TSPAN3 resulted in upregulated expression of β1 integrin, which can be arrested by the deletion of LEL domain in TSPAN3-ΔLEL, further suggesting that LEL domain is required for the upregulation of β1 integrin by TSPAN3 (Fig. 2F).

We further validated the involvement of TSPAN3 in the elevated expression of β1-integrin through IHC in 20 NSCLC samples randomly selected from the 81 tissues. Correlation analysis revealed that tissues with high levels of TSPAN3 also had high levels of β1 integrin, whereas those with low levels of TSPAN3 had low levels of β1 integrin (Fig. 2G), indicating a positive correlation between the expression of TSPAN3 and β1 integrin (*P* = 0.022, Table 2).

**Table 2:** Correlation between tetraspanin 3 (TSPAN3) expression and β1 integrin levels in patients with non-small cell lung cancer

		β1 integrin		Chi-square test	
		Negative	Positive	*χ*^2^	*P*
TSPAN3	Negative	5	2	6.282	0.022
	Positive	2	11		

### TSPAN3 activates the FAK/MAPK pathway and promotes NSCLC cell proliferation via β1 integrin

**Fig. 3:**
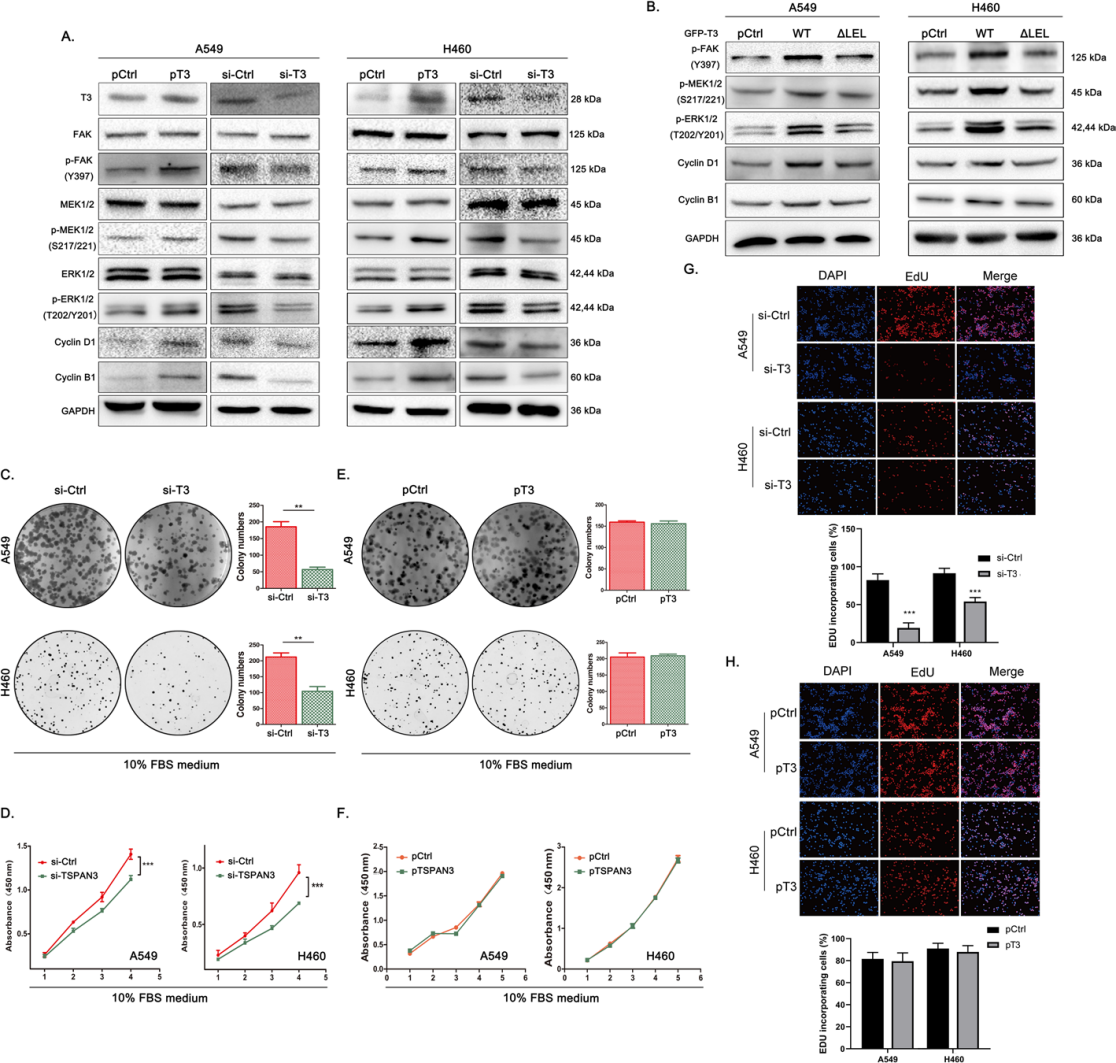
Effect of TSPAN3 expression on the proliferation of NSCLC cell lines in vitro without the presence of FN. **A**Western blot showing that overexpression of tetraspanin 3 (TSPAN3/T3) in A549 cells results in increased phosphorylation of FAK, MEK, and ERK. Knockdown of TSPAN3 in H460 cells exhibited an opposite effect. GAPDH served as the loading control (*n* = 3 independent experiments). **B** Augmented phosphorylation of FAK, MEK, and ERK, and upregulation of cyclin B1 and cyclin D1 mediated by WT TSPAN3 was abrogated by LEL domain deletion (ΔLEL). GAPDH served as the loading control (*n* = 3 independent experiments). **C-E** Colony formation, MTS assays and Edu assays showed inhibited proliferation of A549 and H460 cells in the context of TSPAN3 downregulation (*n* = 3 independent experiments). **F–H** TSPAN3 overexpression failed to promote the proliferation of A549 and H460 cells, as detected by colony formation MTS assays and Edu assays (*n* = 3 independent experiments). *P < 0.05; **P < 0.01; ***P < 0.001

Next, the effects of TSPAN3 overexpression and knockdown on the phosphorylation of key FAK/MAPK pathway intermediaries downstream to β1 integrin activation [23] were investigated. Overexpression of TSPAN3 resulted in significantly increased phosphorylation of FAK, MEK, and ERK, whereas overexpression of the LEL-deleted TSPAN3 mutant did not exhibit a similar effect. This finding further demonstrates that TSPAN3 activates the FAK/MAPK signaling pathway through LEL-mediated interaction with β1 integrin. Conversely, TSPAN3 knockdown resulted in reduced phosphorylation of these key factors (Fig. 3A, B).

**Fig. 4:**
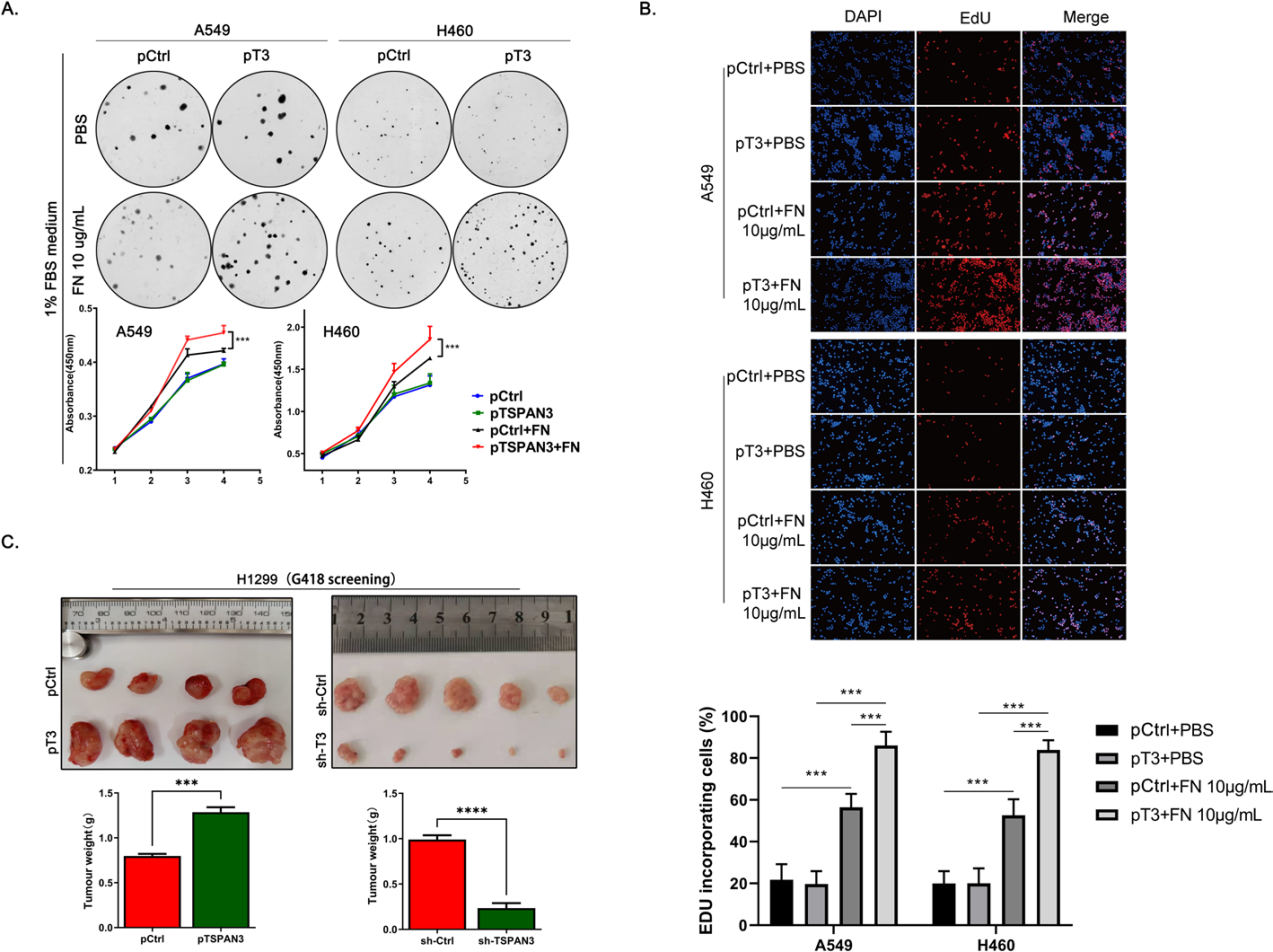
Effect of TSPAN3 overexpression on the proliferation of NSCLC cell lines in vitro with the presence of FN and in vivo. **A**, **B** TSPAN3 overexpression promoted the proliferation of A549 and H460 cells in presence of fibronectin (FN), as detected by colony formation, MTS and Edu assays (*n* = 3 independent experiments). **C** The volume and weight of transplanted tumors in mice injected with cells stably overexpressing TSPAN3 were greater than those in mice injected with the empty vector, whereas the transplanted tumors in mice injected with cells stably knockdown of TSPAN3 showed opposite effects [pTSPAN3 versus pControl (pCtrl), volume: 0.51 ± 0.09 cm^3^ versus 1.38 ± 0.13 cm^3^ (*P* < 0.01), weight: 0.80 ± 0.05 g versus 1.29 ± 0.11 g (*P* < 0.001); sh-TSPAN3 versus sh-control (sh-Ctrl), volume: 0.01 ± 0.01 cm^3^ versus 0.61 ± 0.37 cm^3^ (*P* < 0.05), weight: 0.23 ± 0.12 g versus 0.99 ± 0.11 g (*P* < 0.0001]]. *P < 0.05; **P < 0.01; ***P < 0.001;****P < 0.0001. FBS, bovine serum; PBS, phosphate buffered saline

The function of TSPAN3 in NSCLC cell proliferation was further explored. Colony formation, MTS assays, and Edu assays revealed that TSPAN3 knockdown reduced the proliferation of A549 and H460 cells (Fig. 3C–E), whereas overexpression of TSPAN3 in these cells showed negligible impact on proliferation in both cell lines (Fig. 3F–H). We speculate that this lack of progrowth effect is due to the shortage of access to integrin ligands in our in vitro culture that are otherwise highly present in vivo. Therefore, A549 and H460 cells were treated with FN (10 μg/mL) as an integrin ligand. As expected, in the presence of FN, TSPAN3 overexpression promoted the proliferation of these NSCLC cells (Fig. 4A, B). Furthermore, variations in TSPAN3 levels consistently affected the expression of proteins involved in cell proliferation. Specifically, the expression of cyclin D1 and cyclin B1 was upregulated following TSPAN3 overexpression, and overexpression of the TSPAN3-ΔLEL mutant abrogated these effects. Conversely, TSPAN3 knockdown resulted in the decreased expression of cyclin D1 and cyclin B1 (Fig. 3A, B).

The phosphorylation of FAK, MEK, ERK and level of cyclinB1, cyclinD1 increased after TSPAN3 upregulation without the presence of FN (Fig. 3A, B), which is inconsistent with the results of Colony formation, MTS assays, and Edu assays. We attribute this contradiction to the difference in time point of measurement. Increased phosphorylation of FAK, MEK, ERK and level of cyclinD1, cyclinB1 were detected at 48 h after transfection of TSPAN3 overexpression plasmid, when increased TSPAN3 caused a steady increased level of β1 integrin, resulting in the detectable transient increased in the downstream signaling pathways and cyclinD1 and cyclinB1. In contrast, the cells were continuously observed for a prolonged culture period until 144 h for colony formation, MTS assays, and Edu assays without the presence of FN, when increased level of β1 integrin no longer sustained the activation of downstream signaling pathway or levels of cyclinB1 and cyclinD1. Therefore, without FN stimulation, the enhanced proliferation in NSCLC cells was not observed in colony formation, MTS assays, and Edu assays after TSPAN3 overexpression. To prove this hypothesis, we further detected the phosphorylation of FAK, MEK, and ERK, as well as the level of cyclinD1 and cyclinB1 at multitime points after the transfection of TSPAN3 overexpression plasmid with or without FN. As the results indicated, increased level of β1 integrin was always observed through 120 h post transfection regardless of FN treatment, whereas the upregulation in phosphorylation of FAK, MEK, ERK, and the level of cyclinD1 and cyclin B1 began to attenuate at 72 h post transfection in the absence of FN. In contrast, with the stimulation of FN, the upregulation in phosphorylation of FAK, MEK, ERK, and the level of cyclinD1 and cyclin B1 was sustained until 120 h post transfection (Supplementary Fig. 1).

To further explore the effects of TSPAN3 on NSCLC proliferation in vivo, H1299 cells stably transfected with the TSPAN3-overexpressing plasmid or the sh-TSPAN3 short hairpin RNA (selected by G418, Supplementary Fig. 2) were subcutaneously delivered into nude mice for the assessment of tumor formation. Tumor volume and weight in the TSPAN3-overexpressing group were significantly higher than those in control group. In contrast, knockdown of TSPAN3 showed opposite impact (total of five nude mice per group). (Fig. 4C). All these data suggest that TSPAN3 functions as a promoter of NSCLC progression in vitro and in vivo.

### TSPAN3 promotes β1 integrin recycling in NSCLC cells

**Fig. 5:**
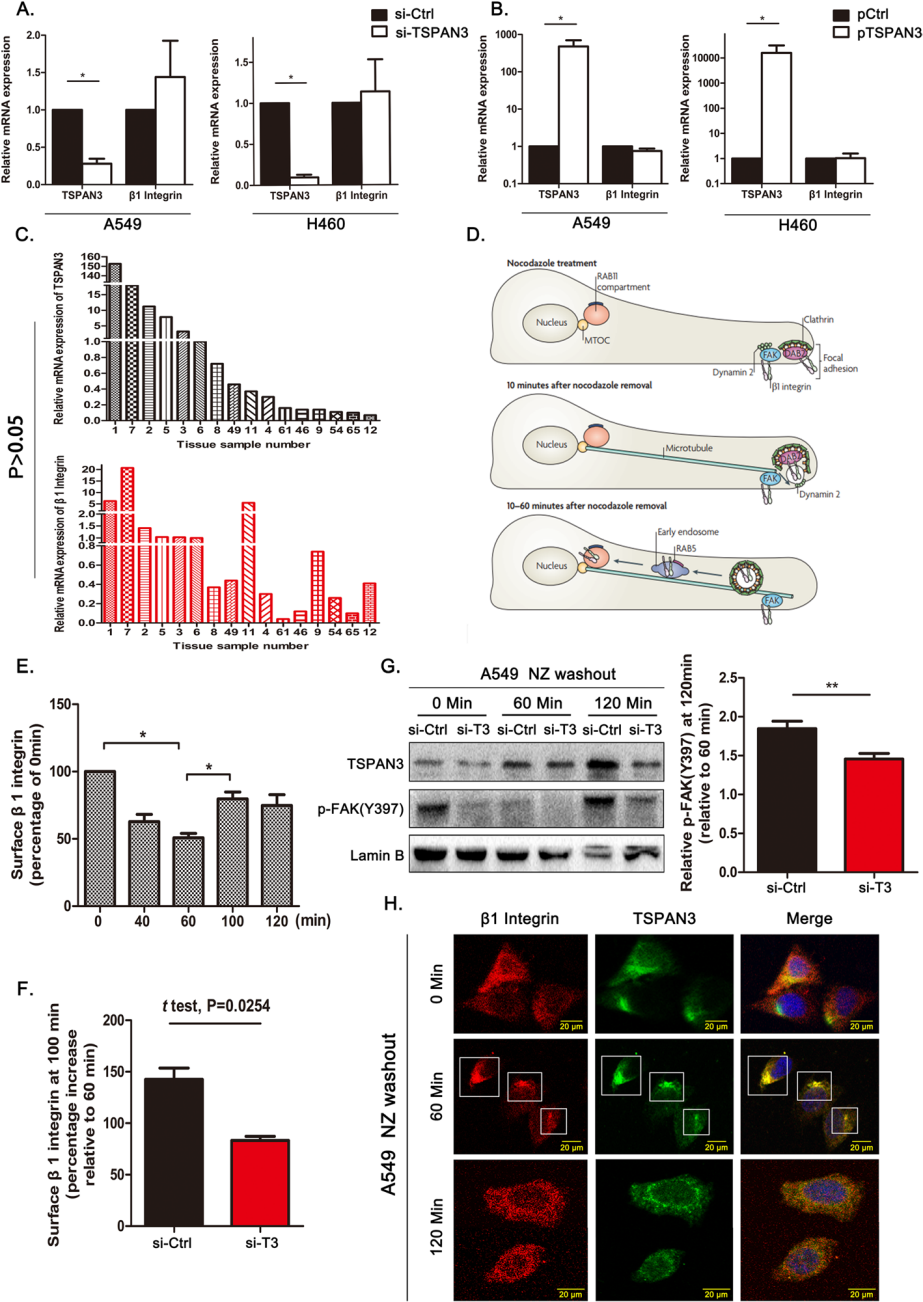
Impact of TSPAN3 expression on the intracellular recycling of β1 integrin. **A**, **B** β1 integrin (*ITGB1*) mRNA levels were detected through RT-PCR in A549 and H460 cells with tetraspanin 3 (TSPAN3/T3) knockdown and overexpression (*n* = 3 independent experiments). **C** Relative mRNA expression of *TSPAN3* and *ITGB1* in 16 NSCLC samples as analyzed by RT-PCR. The 2^−ΔΔCt^ value of each sample was calculated with the no. 6 sample as the reference. (*P* > 0.05).** D** Model depicting the cellular response associated with nocodazole (NZ) washout [2].** E** β1 integrin expression on the surface of A549 cells at the indicated time points after NZ washout was assessed by flow cytometry. The expression values were normalized to those at 0 min (*n *= 3 independent experiments).** F** Surface expression of β1 integrin at 100 min after NZ washout relative to that at 60 min after NZ washout in A549 cells transfected with either si-TSPAN3 or si-Ctrl (*n* = 3).** G** The levels of p-FAK at different time points after NZ washout. The levels of p-FAK at 100 min after NZ washout relative to that at 60 min were calculated after normalizing to Lamin B levels (right, *n* = 3 independent experiments).** H** Immunofluorescence depicting the localization of TSPAN3 (TRITC) and β1 integrin (FITC) in A549 cells at different time points after NZ washout. Magnification ×400. *P < 0.05; **P < 0.01; ***P < 0.001

To investigate the mechanism by which TSPAN3 regulates β1 integrin production, β1 integrin (*ITGB1*) messenger RNA levels were first measured. PCR analysis revealed that neither TSPAN3 overexpression nor its knockdown significantly affected *ITGB1* expression (Fig. 5A, B). Moreover, PCR analysis of 16 NSCLC samples showed no positive correlation between *TSPAN3* and *ITGB1* expression (Fig. 5C), suggesting that TSPAN3 influences β1 integrin production at the post-translational level. Considered of this finding and previous studies indicating the capacity of tetraspanins in regulating various aspects of membrane receptor trafficking, it is reasonable to hypothesize that TSPAN3 upregulates β1 integrin production via the regulation of β1 integrin trafficking and recycling. To test our hypothesis, we firstly validated the feasibility of a NZ treatment-based tracing method of integrin trafficking in our study [2, 24, 25]. Our results reveal a time sensitive, detectable restoration of the surface β1 integrin expression to baseline levels 100 min after NZ washout (Fig. 5D, E).

Thus, the NZ washout method was adopted to synchronize endocytosis of β1 integrins and further explore the impact of TSPAN3 on β1 integrin recycling. Flow cytometric analysis indicated that A549 cells lacking TSPAN3 were unable to restore the surface β1 integrin levels 100 min after NZ washout (Fig. 5F). Accordingly, knocking down TSPAN3 in A549 cells resulted in a failure in restoring FAK (Tyr397) phosphorylation to levels similar to those in the control group (100 min after NZ washout; Fig. 5G). Immunofluorescence on A549 cells was performed at different time points after NZ washout to verify the involvement of TSPAN3 in β1 integrin recycling. At 60 min, most of the β1 integrin had been endocytosed and transported to the perinuclear region. At this time point, most of the TSPAN3 had localized to the perinuclear region and exhibited intense colocalization with β1 integrin. At 120 min, most of the β1 integrin had returned to the membrane, and the colocalization of TSPAN3 and β1 integrin was lost (Fig. 5H). Taken together, these data suggest that TSPAN3 is involved in, and promotes, the recycling of β1 integrin.

### TSPAN3 promotes β1 integrin recycling by facilitating its sorting into Rab11a recycling endosomes

**Fig. 6:**
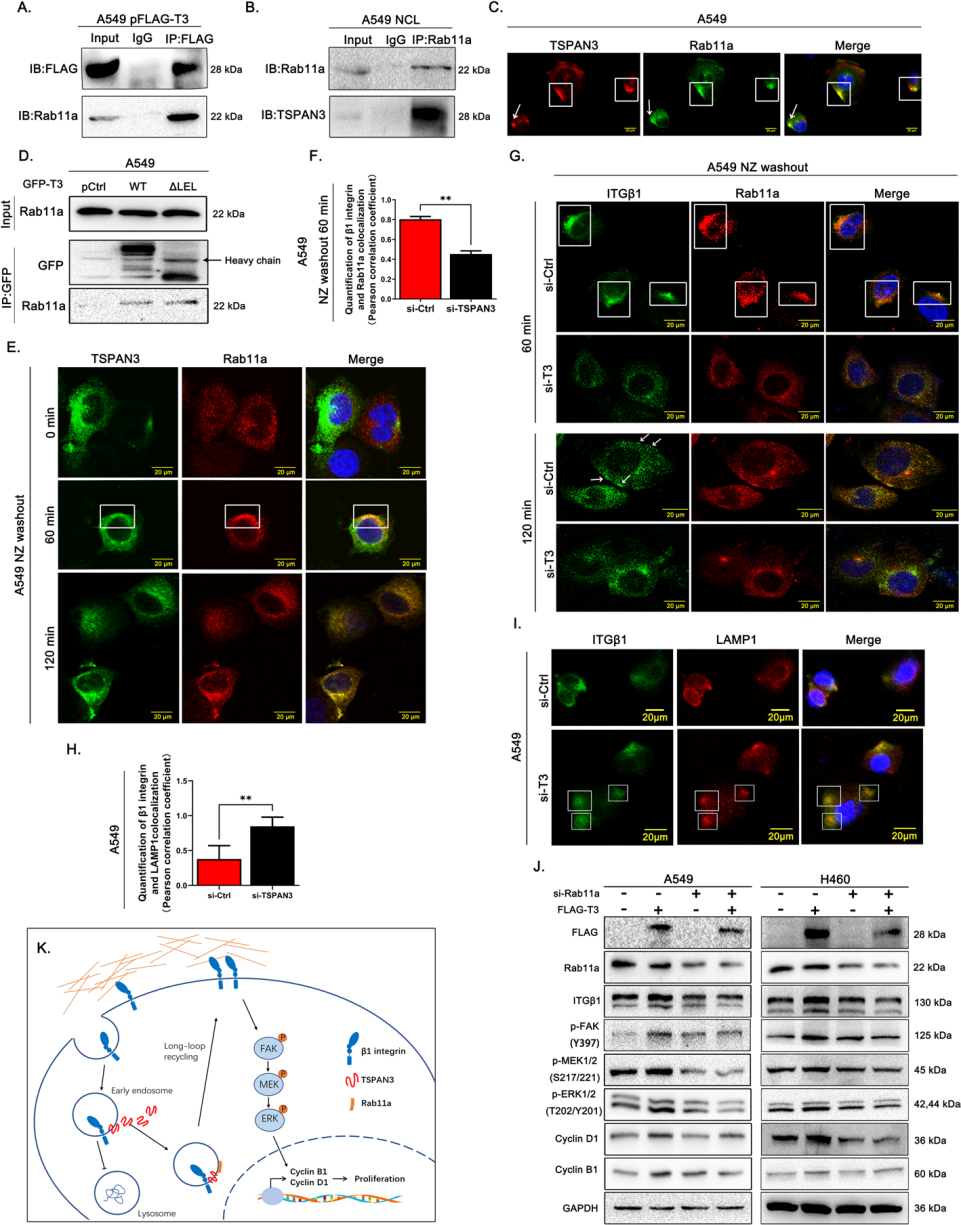
Effect of TSPAN3 on β1 integrin recycling. **A** Coimmunoprecipitation assays showing the interaction between exogenous tetraspanin 3 (TSPAN3/T3) and endogenous Rab11a in A549 cells. A549 cells were transfected with pCMV6-Myc-FLAG-TSPAN3 constructs. After 48 h of transfection, cell lysates were immunoprecipitated with anti-FLAG antibodies or control IgG and subjected to western blotting with anti-Rab11a and anti-FLAG antibodies. **B** Coimmunoprecipitation assay showing the interaction between endogenous TSPAN3 and Rab11a in A549 cells **C** Immunofluorescence showing the localization of TSPAN3 (TRITC) with Rab11a (FITC) in the cytoplasm of A549 cells. Magnification ×400.** D** Western blot indicating that the interaction between TSPAN3 and Rab11a does not depend on the LEL domain of TSPAN3. After 48 h of transfection for wild-type GFP-TSPAN3 or TSPAN3 mutant, cell lysates were immunoprecipitated with the anti-GFP antibody; the presence of Rab11a in the anti-GFP-precipitated complex was examined through western blotting with the anti-Rab11a antibody. ** E** Immunofluorescence showing the colocalization of TSPAN3 (TRITC) and Rab11a (FITC) in A549 cells at different time points after nocodazole (NZ) washout (magnification ×400).** F** Pearson correlation coefficient of the colocalization between Rab11a and β1 integrin at 60 min after NZ washout. **G** Immunofluorescence showed that TSPAN3 knockdown (si-T3) resulted in reduced colocalization of β1 integrin and Rab11a (60 min after NZ washout) and the surface expression of β1 integrin (120 min, indicated by arrowheads). Magnification ×400. **H** Immunofluorescence showed that TSPAN3 knockdown (si-T3) resulted in increased colocalization of β1 integrin and LAMP1 at random time without NZ washout. Magnification ×400. **I** Pearson correlation coefficient of the colocalization between LAMP1 and β1 integrin at random time without NZ washout. **J** Western blot indicating that Rab11a knockdown attenuated the TSPAN3-induced increase in β1 integrin, p-FAK (Tyr397), p-MEK, p-ERK, cyclin B1, and cyclin D1 expression. GAPDH served as the loading control (*n* = 3 independent experiments). **K** Schematic of the mechanism of TSPAN3 regulating β1 integrin intracellular recycling, TSPAN3 promotes β1 integrin intracellular recycling by facilitating β1 integrin sorting into Rab11a recycling endosomes, which reduces β1 integrin lysosomal degradation and enables more rapid resurface of integrin

To further explore the specific mechanism underlying the involvement of TSPAN3 in β1 integrin recycling, mass spectrometric analysis was performed. Consistent with reports that the Rab11-dependent mechanism is one of the major routes for integrin recycling, the mass spectrometric data (Supplementary Figs. 3 and 4) indicated that TSPAN3 could promote β1 integrin recycling via Rab11a. The interaction between TSPAN3 and Rab11a was further verified in A549 cells through coimmunoprecipitation (Fig. 6A, B). Immunofluorescence also revealed the colocalization of TSPAN3 and Rab11a in the cytoplasm (Fig. 6C). Furthermore, this interaction did not depend on the LEL domain of TSPAN3 (Fig. 6D). Interestingly, a dynamic change in the colocalization of TSPAN3 and Rab11a was observed at different time points after NZ washout. At 60 min post NZ washout, TSPAN3 also colocalized with Rab11a, which showed an overlapping pattern with that of the dynamic change of colocalization between TSPAN3 and β1 integrin after NZ washout. The colocalization was maintained up until 120 min after NZ washout (Fig. 6E), Additionally, TSPAN3 knockdown or overexpression did not affect Rab11a levels (Supplementary Fig. 5). Combining previous studies and the present findings, we inferred that TSPAN3 may interact with Rab11a and β1 integrin, thereby facilitating β1 integrin sorting into Rab11a recycling endosomes.

Immunofluorescence on A549 cells at different time points after NZ washout was performed next to explore the changes in the interaction between β1 integrin and Rab11a after TSPAN3 knockdown. The results showed that at 60 min after NZ washout, most of the β1 integrin had been endocytosed in both the si-TSPAN3 and control groups. However, in the control group, the endocytosed β1 integrin showed significant colocalization with Rab11a, whereas the colocalization between β1 integrin and Rab11a was lower after TSPAN3 knockdown (Fig. 6F, G). Correspondingly, at 120 min after NZ washout, most of the β1 integrin in the control group had returned to the periphery of the cell, whereas most of the β1 integrin in the si-TSPAN3 group remained in the cytoplasm (Fig. 6G). After the knockdown of TSPAN3 in A549, increased colocalization of β1 integrin and LAMP-1(the biomarker of lysosome) were found at all time points (Fig. 6H, I). Moreover, immunoblotting showed that si-Rab11a transfection of both A549 and H460 cells attenuated the TSPAN3-induced elevation in β1 integrin, p-FAK, p-MEK, p-ERK, cyclin D1, and cyclin B1 levels (Fig. 6J), which further supported our hypothesis. Based on all above-mentioned findings, we concluded that TSPAN3 promotes β1 integrin recycling, upregulates β1 integrin levels, and promotes NSCLC cell proliferation through a Rab11a-dependent mechanism (Fig. 6K).

## Discussion

To our best knowledge, the biological function and the underlying mechanism of TSPAN3 in NSCLC behavior have not been extensively studied or reported. The results of the present study show that TSPAN3 promotes β1 integrin recycling through interaction with β1 integrin and Rab11a, thereby upregulating β1 integrin levels and further promoting the proliferation of NSCLC.

TSPAN3 has been reported to form a complex with β1 integrin in oligodendrocytes and could promote the proliferation of mouse oligodendrocyte cells [14], which is consistent with the our observations in NSCLC cell lines. Moreover, recent studies have identified key roles for some tetraspanins in the trafficking and functional regulation of membrane proteins, such as β1 integrins [26]. In agreement with these reports, TSPAN3 was found to upregulate β1 integrin by regulating its intracellular recycling.

Intracellular recycling of integrins dictates membrane integrin levels and function. The Rab11-dependent route is the main pathway through which most integrins are transferred from the cytoplasm to the cell membrane [27, 28]. Despite this common turnover route, each integrin subunit is known to have distinct trafficking characteristics, suggesting that sophisticated mechanisms are involved in regulating the transport of specific integrins through certain routes [29]. In this study, TSPAN3 was found to be recruited to Rab11a recycling endosomes in concomitant with integrin sorting into Rab11a endosomes. Furthermore, knockdown of TSPAN3 reduced the colocalization of β1 integrin and Rab11a after β1 integrin endocytosis. Taken together, these findings suggest that TSPAN3 functions as a sorting mediator for β1 integrin recycling though a Rab11-dependent route. Additionally, consistently high levels of TSPAN3 were observed in NSCLC tissues and cell lines. Furthermore, high levels of TSPAN3 positively correlated with poor differentiation, lymph node involvement, advanced pathological tumor-metastasis-node stage, and poor prognosis among patients with NSCLC. Therefore, TSPAN3 may be a tumor promoter in NSCLC.

To our knowledge, this is the first study to report that TSPAN3 acts as a promoter of β1 integrin intracellular recycling by facilitating β1 integrin sorting into Rab11a recycling endosomes, which in turn upregulates β1 integrin levels. These findings provide further understanding of the mechanisms underlying integrin trafficking and provides new avenues for future exploration. For example, TSPAN3 was shown to be recruited into Rab11a endosomes and TSPAN3 trafficking accompanies the regulation of β1 integrin recycling; however, the mechanisms that control these processes remain unknown and would be an interesting subject for further study. The role of LEL domain in the interaction between TSPAN3 and β1 integrins was also herein clarified while the function of other TSPAN3 domains, especially that of the YXXΦ motif in the TM4 domain supposedly involved in vesicle sorting, remains unclear. Likewise, more in-depth mechanistic studies are needed to address the major limitations of our findings: (1) the pattern of direct protein–protein interaction in the TSPAN3-β1-Rab11a axis remains unclear in the absence of GST-pulldown study, and (2) whether TSPAN3 regulates other aspects of β1 integrin trafficking, such as endocytosis and lysosomal degeneration, and whether TSPAN3 regulates the recycling of other membrane receptors, such as the epidermal growth factor receptor, will have to be explored.

In summary, TSPAN3 regulates intracellular recycling of β1 integrin via the Rab11-dependent route. This cascade, in turn, upregulates β1 integrin to promotes NSCLC proliferation. To our knowledge, this is the first study to report this role of TSPAN3. Furthermore, these findings suggest that TSPAN3 is a potential target for drug development in lung cancer.

## Supplementary Information

Supplementary materials 1: **Table S1.**. Antibodies used in the study. **Table S2.** Cox regression analysis of TSPAN3 expression and NSCLC patients’ prognosis. **Figure S1. **Western blot analysis for phosphorylation of FAK, MEK, ERK and the level of CyclinD1, Cyclin B1 at multiple time points after the transfection of TSPAN3 overexpression plasmid in the presence or absence of FN, respectively.** Figure S2. **Transfection efficiency for stably transfection of TSPAN3-overexpressing plasmid or sh-TSPAN3 short hairpin RNA in H1299.** Figure S3. **Mass spectrometric analysis reveals that TSPAN3 can interact with Rab11a.** Figure S4. **Mass spectrometric analysis of Rab11a protein-related peptides.** Figure S5. **Rab11a levels do not vary regardless of TSPAN3 expression.

## Data Availability

The datasets used and/or analyzed during the current study are available from the corresponding author on reasonable request.

## Abbreviations

FBS: Fetal bovine serum
IHC: Immunohistochemistry
IP: Immunoprecipitation
ITG: Integrin
LEL: Large extracellular loop
NSCLC: Non-small cell lung cancer
NZ: Nocodazole
Rab11a: Ras-related protein Rab-11a
RT-PCR: Real-time polymerase chain reaction
SEL: Small extracellular loop
TSPAN3: Tetraspanin 3
