# Supplementary material for: Tetraspanin 3 promotes NSCLC cell proliferation via regulation of β1 integrin intracellular recycling
Source: Cell Mol Biol Lett. 2024 Sep 27;29:124. doi: 10.1186/s11658-024-00639-w (PMC11428915; doi:10.1186/s11658-024-00639-w)
Supplement: Supplementary file 1 — Supplementary materials 1: Table S1.. Antibodies used in the study. Table S2. Cox regression analysis of TSPAN3 expression and NSCLC patients’ prognosis. Figure S1. Western blot analysis for phosphorylation of FAK, MEK, ERK and the level of CyclinD1, Cyclin B1 at multiple time points after the transfection of TSPAN3 overexpression plasmid in the presence or absence of FN, respectively. Figure S2. Transfection efficiency for stably transfection of TSPAN3-overexpressing plasmid or sh-TSPAN3 short hairpin RNA in H1299. Figure S3. Mass spectrometric analysis reveals that TSPAN3 can interact with Rab11a. Figure S4. Mass spectrometric analysis of Rab11a protein-related peptides. Figure S5. Rab11a levels do not vary regardless of TSPAN3 expression. [file 11658_2024_639_MOESM1_ESM.docx]

*Supplementary Material Online*

**Tetraspanin 3 promotes NSCLC cell proliferation via regulation of β1 integrin intracellular recycling**

Yao Zhang^1*^, Chenglong Wang^2*^, Yitong Xu^3^, Hongbo Su^3^

^1^Department of pathology, The First Hospital and Basic Medical Sciences College of China Medical University, Shenyang 110001, China.

^2^Department of Pain, The First Hospital of China Medical University, Shenyang 110001, China.

^3^Department of pathology, The First Hospital of China Medical, The First Hospital of China Medical University, Shenyang 110001, China.

*These authors contributed equally to this work

***Corresponding author**:hbsu@cmu.edu.cn, [ytxu@cmu.edu.cn](mailto:hbsu@cmu.edu.cn)

**Supplementary materials and methods**

**Information on si-RNAs and plasmids used in the study**

TSPAN3- and RAB11a-specific siRNAs and scrambled control siRNAs were purchased from RiboBio (Guangzhou, China). The pCMV6-Myc-FLAG-TSPAN3 construct (#RC203876) and the empty vector were purchased from OriGene (Rockville, MD, USA). The pCMV6-GFP-TSPAN3-ΔLEL construct containing a splice variant (LEL deletion) of wild-type TSPAN3 was purchased from TSINGKE Biological Technology (Beijing, China).

**Primer sequences used in the study**

TSPAN3 forward, 5′–ATGGAACCAACCCTGATGCTGCTAG–3′;

TSPAN3 reverse, 5′–AGTCTCTCTGCAGCAGCTAAGAGGG–3′;

β1 integrin forward: 5′–CAAGAGAGCTGAAGACTATCCCA-3′;

β1 integrin reverse: 5′-TGAAGTCCGAAGTAATCCTCCT–3′;

β-actin forward: 5′–ATAGCACAGCCTGGATAGCAACGTAC-3′;

β-actin reverse: 5′–CACCTTCTACAATGAGCTGCGTGTG–3′.

**Tumour formation in nude mice**

Nude mice used in this study were treated following the experimental animal ethics guidelines issued by the China Medical University. Four-week-old female BALB/c nude mice were purchased from Slac (Shanghai, China) and were maintained in a laminar-flow cabinet under specific pathogen-free conditions for one week before use. Each mouse was inoculated subcutaneously in the right axilla with 1 × 10^7^ tumour cells (selected by G418) in 0.2 mL sterile phosphate-buffered saline. Four weeks after axilla inoculation, the mice were euthanized, and necropsies were performed to examine tumour growth.

**Proliferation Edu Assay**

Cells transfected with TSPAN3-expressing plasmid or empty vector were seeded (500 cells/well) in 6-well and incubated for 24 hours. The EdU analysis kit (Beyotime Biotechnology, CAT#C0075S) was used to analyse and evaluate cell proliferation, and the specific method was carried out following the instructions provided by the manufacturer. Images were captured using an Olympus FV3000 laser-scanning confocal microscope (Olympus, Tokyo, Japan).

| **Table S1.** Antibodies used in the study | | | | |
| --- | --- | --- | --- | --- |
| **Antibody name** | **Company** | **Catalogue No.** | **Host** | **Working dilution** |
| TSPAN3 | Abcam | ab151299 | Rabbit | WB: 1:500 |
| TSPAN3 | Thermo Fisher Scientific | PA5-28514 | Rabbit | WB: 1:500  IF: 1:50 |
| TSPAN3 | Thermo Fisher Scientific | PA5-25472 | Rabbit | WB: 1:500  IHC: 1:100 |
| GFP tag | TransGen Biotech | HT801 | Mouse | WB: 1:500 |
| GFP tag | Proteintech | 50430-2-AP | Rabbit | WB: 1:500 |
| FLAG tag | TransGen Biotech | HT201-01 | Mouse | WB: 1:500 |
| RAB11a | Proteintech | 20229-1-P | Rabbit | WB: 1:1,000 |
| RAB11a | Santa Cruz Biotechnology | sc166523 | Mouse | WB: 1:200  IF: 1:50 |
| β1 integrin | Santa Cruz Biotechnology | sc59829 | Mouse | WB: 1:200 |
| β1 integrin | Proteintech | 26918-1-AP | Rabbit | WB: 1:500  IF: 1:50  IHC: 1:100 |
| Cyclin B1 | Cell Signaling Technology | 4138 | Rabbit | WB: 1:1,000 |
| Cyclin D1 | Cell Signaling Technology | 32922 | Rabbit | WB: 1:1,000 |
| MEK1/2 | Wanleibio | WL03328 | Rabbit | WB: 1:500 |
| p-MEK1/2 | Cell Signaling Technology | 9154 | Rabbit | WB: 1:1,000 |
| ERK1/2 | Cell Signaling Technology | 4695 | Rabbit | WB: 1:1,000 |
| p-ERK1/2 | Cell Signaling Technology | 9101 | Rabbit | WB: 1:1,000 |
| FAK | Cell Signaling Technology | 3285 | Rabbit | WB: 1:1,000 |
| p-FAK | Cell Signaling Technology | 8556 | Rabbit | WB: 1:1,000 |
| GAPDH | Proteintech | 10494-1-AP | Mouse | WB: 1:10,000 |
| β-actin | Proteintech | 20536-1-AP | Mouse | WB: 1:10,000 |

| **Table S2.** Cox regression analysis of TSPAN3 expression and prognosis of NSCLC patients | | | | | | | | |
| --- | --- | --- | --- | --- | --- | --- | --- | --- |
| Omnibus Tests of Model Coefficients | | | | | | | | |
| Step(T) | −2 Log Likelihood | Overall (score) | | |  | Change from Previous Block | | |
|  |  | Chi-square | df | P-value |  | Chi-square | df | P-value |
| 1 | 46.360 | 6.035 | 1 | 0.014 |  | 5.858 | 1 | 0.016 |
| a. Beginning Block Number 0. The Original Log-likelihood Function: −2 Log Likelihood: 52.218  b. Beginning Block Number 1. Method = Forward Stepwise (Likelihood Ratio) | | | | | | | | |

| Variables in the Equation | | | | | | | | | |
| --- | --- | --- | --- | --- | --- | --- | --- | --- | --- |
| Step (1) | B | SE | Wald | df | P-value | Exp(B) | 95.0% Exp(B) for CL | | |
|  |  |  |  |  |  |  | Lower |  | Upper |
| TSPAN3 expression | 2.213 | 1.096 | 4.078 | 1 | 0.043 | 9.139 | 1.067 |  | 78.248 |

**
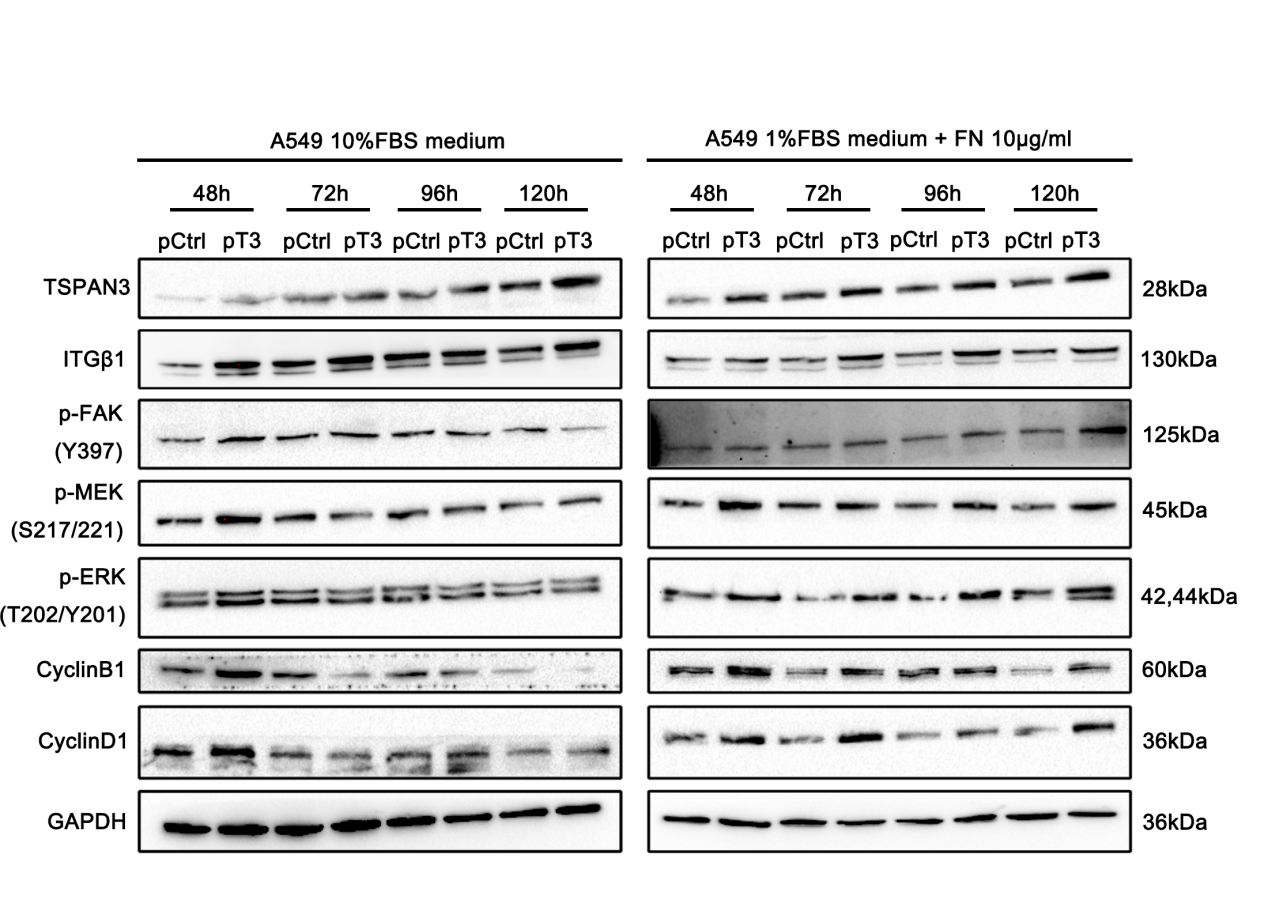
**

**Figure S1. Western blot analysis for phosphorylation of FAK, MEK, ERK and the level of CyclinD1, Cyclin B1 at multiple time points after the transfection of TSPAN3 overexpression plasmid in the absence or presence of FN, respectively. (A)** Immunoblot showing increased level of β1 integrin through 120h post-transfection in the absence of FN, whereas the upregulation in phosphorylation of FAK, MEK, ERK and the level of cyclinD1, cyclin B1 began attenuating at after 72h transfection without the stimulation of FN (n = 3 independent experiments). **(B)** Immunoblot showing steady increased level of β1 integrin stimulated by FN through 120h post-transfection. In the presence of FN, a prolonged upregulation in phosphorylation of FAK, MEK, ERK and the level of cyclinD1, cyclin B1 was observed until 120h after transfection (n = 3 independent experiments).

**
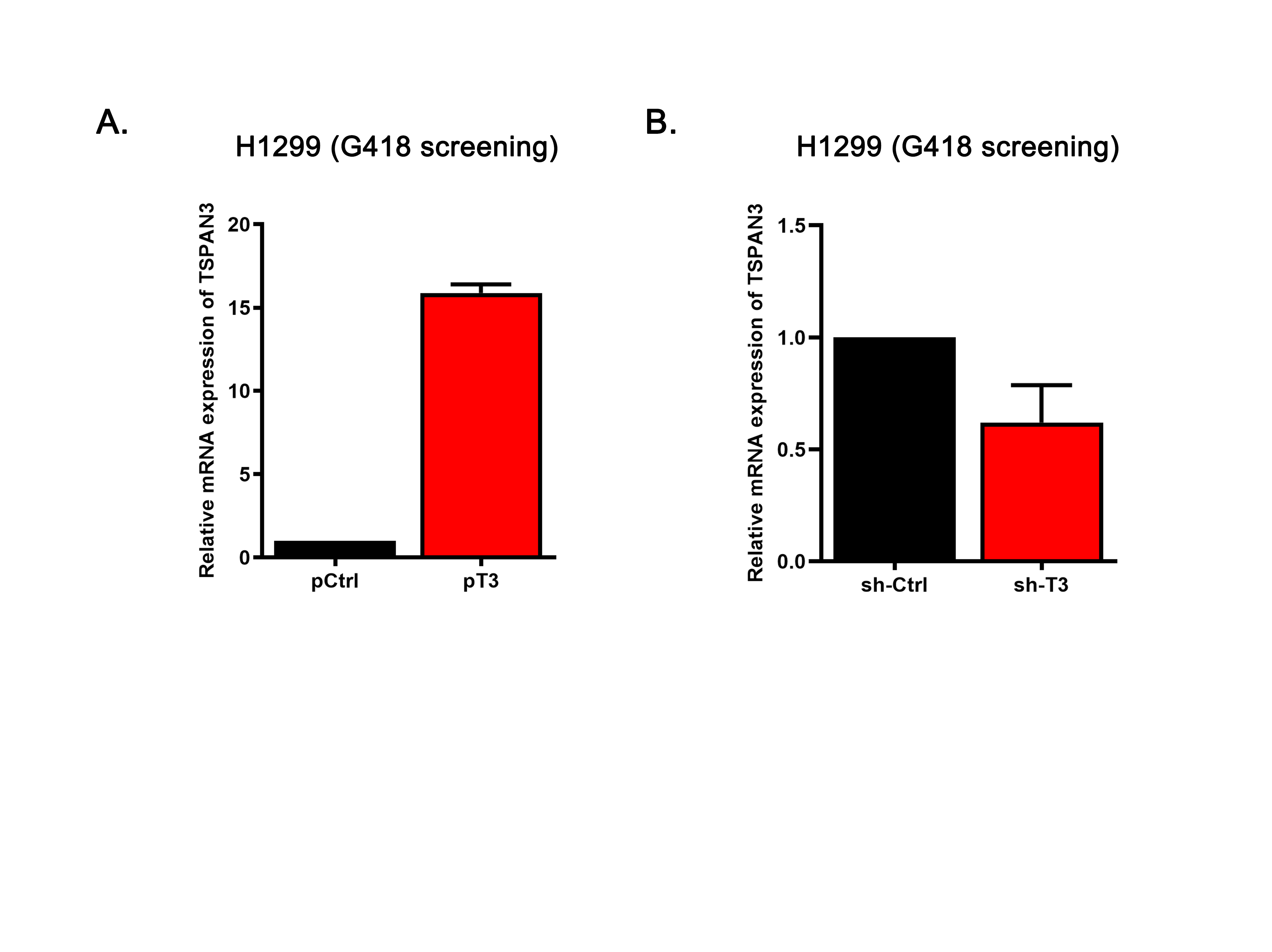
**

**Figure S2. Transfection efficiency for stably transfection of TSPAN3-overexpressing plasmid or sh-TSPAN3 short hairpin RNA in H1299.** TSPAN3 mRNA levels were detected through RT-PCR in H1299 stably transfected with the **(A)** TSPAN3-overexpressing plasmid or the **(B)** sh-TSPAN3 short hairpin RNA (selected by G418)


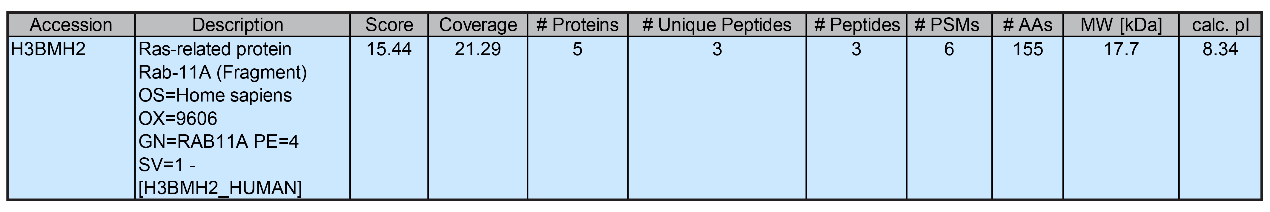


**Figure S3.** Mass spectrometric analysis reveals that TSPAN3 interacts with Rab11a.


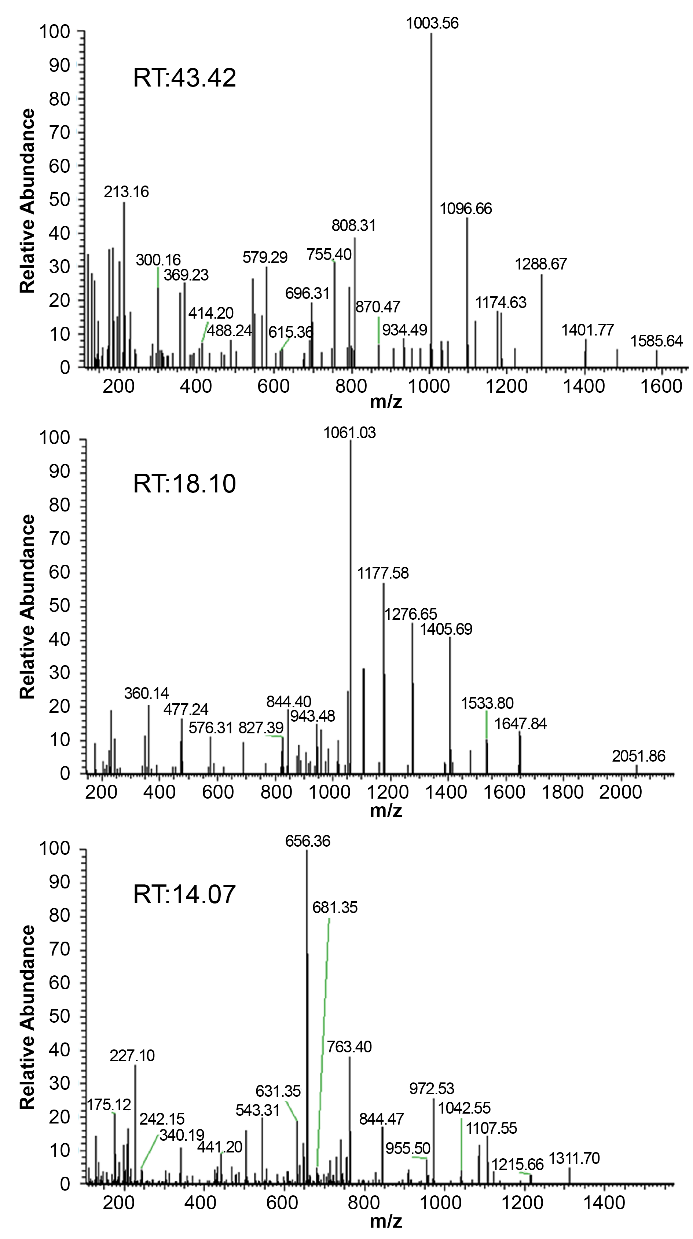


**Figure S4.** Mass spectrometric analysis of Rab11a-related peptides.


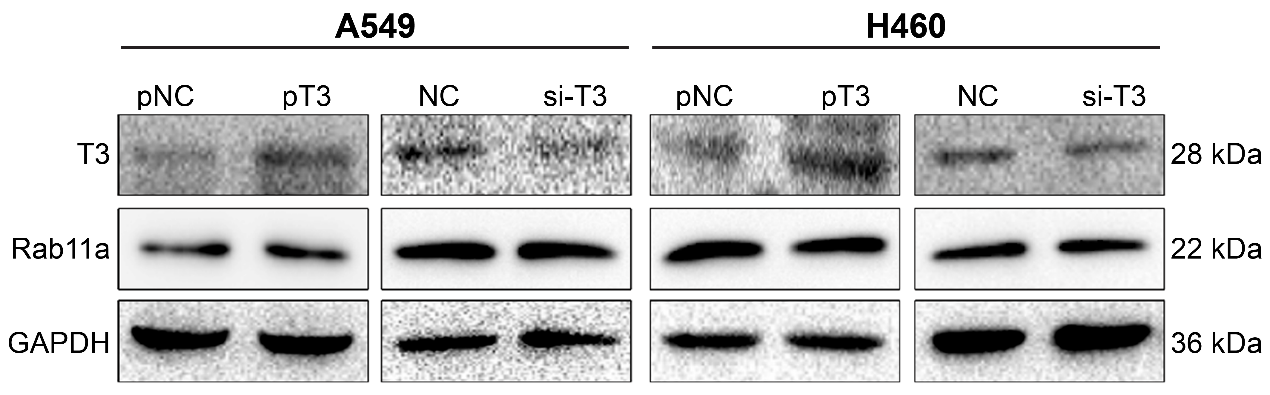


**Figure S5.** Immunoblot showing negligible change in the Rab11a levels regardless of TSPAN3 knockdown (si-T3) or overexpression (pT3) (compared to control A549 and H460 cells (pCtrl/si-Ctrl). GAPDH served as the loading control (n = 3 independent experiments).
